# Evaluation of post-operative foveal location and microstructural changes after pars plana vitrectomy for rhegmatogenous retinal detachment using enhanced-depth imaging optical coherence tomography

**DOI:** 10.1186/s40942-024-00609-6

**Published:** 2024-11-21

**Authors:** Mostafa Mahmoud Eid Al Azaizy, Hossam Eldin Mohamed Khalil, Mahmoud Leila, Nour Salah Akl, Sahar Ibrahim Mohammed

**Affiliations:** 1 Retina Department, Research Institute of Ophthalmology, 2, Al-Ahram Street, Giza, Egypt; 2 Ophthalmology Department, Faculty of Medicine, Beni-Suef University, Beni Suef, Egypt

**Keywords:** Foveal displacement after retinal reattachment, Papillo-foveal distance after retinal detachment surgery, Metamorphopsia post-retinal detachment surgery, Foveal microstructure after retinal detachment surgery

## Abstract

**Background:**

Patients who had successful rhegmatogenous retinal detachment (RRD) surgery often complained of metamorphopsia due to postoperative fovea displacement and alteration of the foveal microstructure. The papillo-foveal distance (PFD) is correlated bilaterally. Therefore, PFD from the fellow healthy eye could be used to determine the change of foveal position in eyes with successful RRD repair. Ultra-high-resolution optical coherence tomography (UHR-OCT) could explain incomplete visual recovery by demonstrating foveal misalignment and changes in foveal microstructure. The rationale of the study is to assess the changes in the foveal location and microstructural layers after successful retinal reattachment and correlate them with visual dysfunction.

**Patients and methods:**

A prospective interventional cross-sectional controlled study included patients who had successful retinal reattachment and complained of defective vision or metamorphopsia. The primary outcome measure is to evaluate the post-operative foveal location. The secondary outcome measures are the assessment of metamorphopsia, the evaluation of the foveal microstructural changes, and the correlation between foveal shift, metamorphopsia, foveal microstructure, and visual function. We used a standard Amsler chart to detect subjective metamorphopsia and a modified Amsler chart to quantify metamorphopsia. We used the enhanced-depth imaging optical coherence tomography (EDI-OCT) to detect changes in PFD and the foveal microstructure. *p* < 0.05.

**Results:**

The study included 50 study eyes and 50 control eyes. The male gender constituted 70%. The mean age was 53 years. The mean baseline BCVA was 0.001. The incidence of foveal displacement was 70%. Disorganized retinal inner layers (DRIL) occurred in 56% of eyes, and disorganized retinal outer layers (DROL) occurred in 72% of eyes. The mean postoperative BCVA was 0.3. The subjective metamorphopsia was mild in 39%, moderate in 24%, severe in 33%, and very severe in 3% of eyes. The mean quantitative metamorphopsia was 587 mm. PVR correlated significantly with the foveal shift. DROL correlated significantly with subjective metamorphopsia. There was a statistically significant difference between subjective metamorphopsia and quantitative metamorphopsia.

**Conclusion:**

Foveal displacement and metamorphopsia after successful retinal reattachment pose significant morbidity. UHR-OCT is pivotal in evaluating the anatomical outcome after successful retinal re-attachment surgery and its relation to visual function.

## Background

The rhegmatogenous type is the most common form of retinal detachment, with an annual incidence of 20 in 100.000. In rhegmatogenous retinal detachment (RRD), fluid ingresses from the vitreous cavity to the subretinal space through a retinal break, separating the neurosensory retina from the underlying retinal pigment epithelium (RPE). In RRD involving the fovea, apoptosis plays an important role in the time-dependent photoreceptor cell damage following retinal detachment. It could lead to profound loss of vision in the affected eye due to loss of photoreceptor outer segments [1]. Microincision vitreoretinal surgery improved RRD’s anatomical and functional success, namely, retinal re-attachment and best-corrected visual acuity (BCVA). Current reported retinal re-attachment rates using modern vitrectomy techniques are 93–97% of cases [2]. Nevertheless, patients who had successful RRD repair often complained of metamorphopsia, aniseikonia, and impaired stereopsis, which significantly compromised the visual function of the patient [3]. The incidence of these co-morbidities ranges from 20 to 49% and they are attributed to misalignment of the fovea relative to its original location and alteration of foveal microstructural layers [4, 5]. The mean papillo-foveal distance (PFD) in normal individuals is 4.76 mm. This distance is highly correlated between both eyes of the same individual. Therefore, PFD from the fellow eye could be used as a reliable and reproducible parameter to determine the change of foveal position in eyes, which had successful RRD repair [6–8]. The recently introduced ultra-high-resolution optical coherence tomography (UHR-OCT) provided insight into the retinal microstructure in an unprecedented resolution and allowed depiction of the pathological changes ensuing in the retinal layers in detached retina and following repair. Hence, UHR-OCT findings could help unravel the discrepancy between successful anatomical reattachment and incomplete visual recovery by demonstrating foveal misalignment and changes in foveal microstructure. [9, 10] The rationale of the present study is to assess whether the change in foveal location and microstructural layers after successful retinal reattachment following pars plana vitrectomy (PPV) for RRD correlates with visual dysfunction.

## Patients and methods

This is a prospective interventional cross-sectional controlled study. The study included 50 consecutive patients, who had successful retinal reattachment after PPV for fovea-involving RRD during 2022. All the included patients complained of defective vision or metamorphopsia. The normal contralateral eye served as the control. The study excluded patients with high myopia and posterior staphyloma, and concurrent retinal or optic nerve diseases that could confound the assessment of the foveal location, or the visual function. These included recurrent retinal detachment, concurrent macular diseases such as macular hole (MH), choroidal neovascularization (CNV), vitreomacular traction (VMT), or macular edema secondary to retinal vascular disorders or uveitis, and congenital anomalies of the optic disc, optic neuropathies, glaucomatous cupping or unequal optic disc cup between both eyes > 0.2. In addition, the study excluded patients who developed postoperative vitreomacular interface (VMI) abnormalities such as epiretinal membrane (ERM) causing foveal distortion or had retinal detachment surgery in the contralateral eye or if the exclusion criteria in the study eye were bilateral. The study excluded any patient with media opacity that precluded sufficient OCT image quality. The primary outcome measure is the evaluation of the post-operative foveal location after successful retinal reattachment surgery. The secondary outcome measures are the assessment of metamorphopsia, the evaluation of the foveal microstructural changes, and the correlation between foveal shift, metamorphopsia, foveal microstructure, and visual function. All recruited patients had a full ophthalmic examination that included detailed history, BCVA measurement using decimal units, intraocular pressure (IOP) measurement using Goldmann’s applanation tonometry, slit-lamp anterior segment examination, dilated fundus examination using slit-lamp biomicroscopy with + 90D lens and indirect ophthalmoscopy using + 20D lens and scleral indentation to assess the detachment extent, number and location of the retinal breaks, grading of PVR, evaluation of the optic disc and the macula in the study eye, and to assess the macula, the optic disc, and the retinal periphery in the control eye. We performed preoperative OCT imaging in the control eye only. Surgical intervention for all patients consisted of 3-port-23gauge PPV using a wide-angle non-contact viewing system (Resight 700 Carl Zeiss Meditec AG, Jena, Germany) and the Constellation Vision System (Alcon Laboratories, Inc., Fort Worth, TX USA). Two qualified vitreoretinal surgeons (ML and MMEA) performed the surgery for RRD in a retinal tertiary center. In case the patient had dense cataracts that hindered retina visualization, the surgeon performed phacoemulsification and intraocular lens implantation (IOL) in the capsular bag before vitrectomy. PPV consisted of core vitrectomy, triamcinolone-assisted induction of PVD, and a 360˚ removal of the peripheral vitreous gel including shaving of the vitreous base. The surgeon then performed fluid-air exchange, and endo-laser retinopexy to the break(s). Additional surgical maneuvers including the application of an encircling scleral band, retinotomy, retinectomy, cryotherapy, use of PFCL, and choice of the type of intra-ocular tamponade were performed at the discretion of the surgeon. The retina was successfully re-attached in all cases intra-operatively. Post-operatively, we instructed all patients to commence strict positioning so that the retinal break was uppermost. The post-operative evaluation included BCVA, IOP measurement, dilated fundus examination, assessment of metamorphopsia using the Amsler grid, and OCT imaging. We performed the latter 2 assessments at 1 month of follow-up in silicone-filled eyes and when sufficient absorption of gas or air tamponade allowed clear media for good-quality images and reliable testing for metamorphopsia.

### Assessment of metamorphopsia

**Fig. 1:**
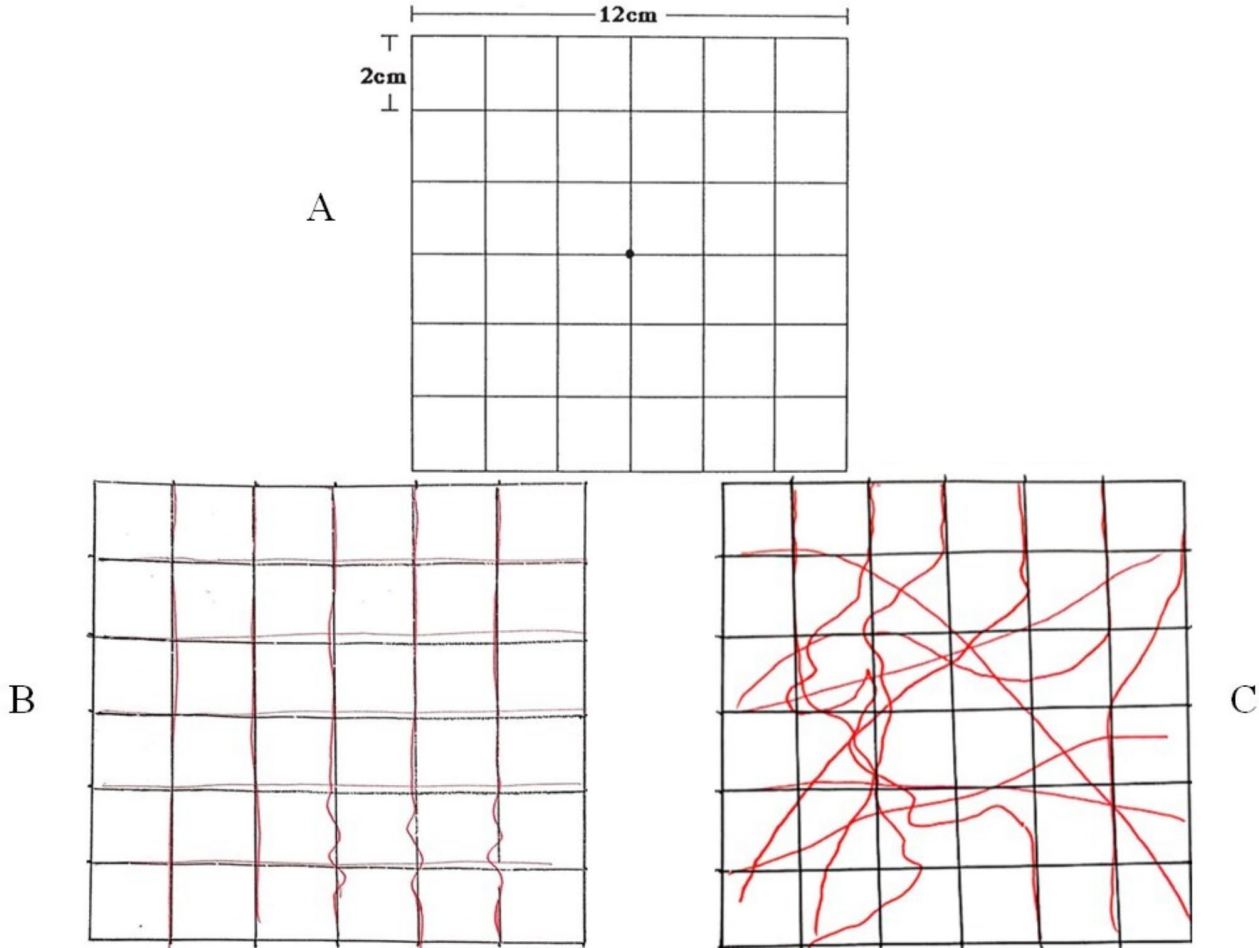
**A**. Modified Amsler chart. **B**. An example of severe quantitative metamorphopsia. The length of all curved or irregular lines the patient drew is 9 cm on the grid. The map scale is 1:100. Thus, the patient score was 900. **C**. An example of a non-gradable quantitative metamorphopsia assessment test. Note the bizarre shapes and irregular lines drawn by the patient

We used a standard Amsler chart for the detection of subjective metamorphopsia. The test consisted of a print-out module held by the patient at 30 cm while wearing the best-corrected refraction and using each eye separately. The patient had to determine whether wavy or curved lines were present. We instructed the patient to rate the severity of metamorphopsia on a scale from 1 to 5 as follows, 1: mild, 2: moderate, 3: severe, 4: very severe, and 5: cannot be assessed. The number that the patient selected represented the subjective metamorphopsia score. We used a modified Amsler chart to quantify metamorphopsia [11]. The chart is a 12 × 12 cm grid comprised of 2 × 2 cm squares. The patient focuses on the center using each eye separately with near vision correction, and while holding the chart 30 cm away. If irregular lines were noticed with the diseased eye, they were traced with the fellow eye. We instructed the patient not to draw any line twice. The length of these lines, excluding those on the outer borders of the grid, was measured on a 1:100 scale (12 cm = 1200 mm). Average line measurements determined the total metamorphopsia value, with higher values indicating worse symptoms. Horizontal and vertical metamorphopsia values were calculated separately. Metamorphopsia severity was categorized based on quantitative measurements as follows, mild metamorphopsia (range: 100 to 399 mm), moderate metamorphopsia (range: 400 to 799 mm), severe metamorphopsia (range: 800 to 1000 mm), and very severe metamorphopsia (range: 1001 to 1200 mm). Cases involving bizarre or irregular drawings were considered ungradable. Figure 1.

### OCT imaging

We performed OCT imaging using the enhanced-depth imaging optical coherence tomography (EDI-OCT); Spectralis HRA + OCT2 (Heidelberg Engineering, Heidelberg, Germany). The OCT imaging protocol consisted of volume scans (25 volume scans with 25 A-scans, 9.2 mm in length), and radial scans (6 radial scans with 30 A-scans each, 9.2 mm in length) using a standard lens (30º lens). We used the wide-view lens of the Heidelberg OCT machine (55° lens) during the measurement of PFD. The assessment of the foveal microstructures included; VMI changes, central macular thickness (CMT), disorganized inner retinal layer (DRIL), and disorganized retinal outer layer (DROL).

#### Measurement of PFD

**Fig. 2:**
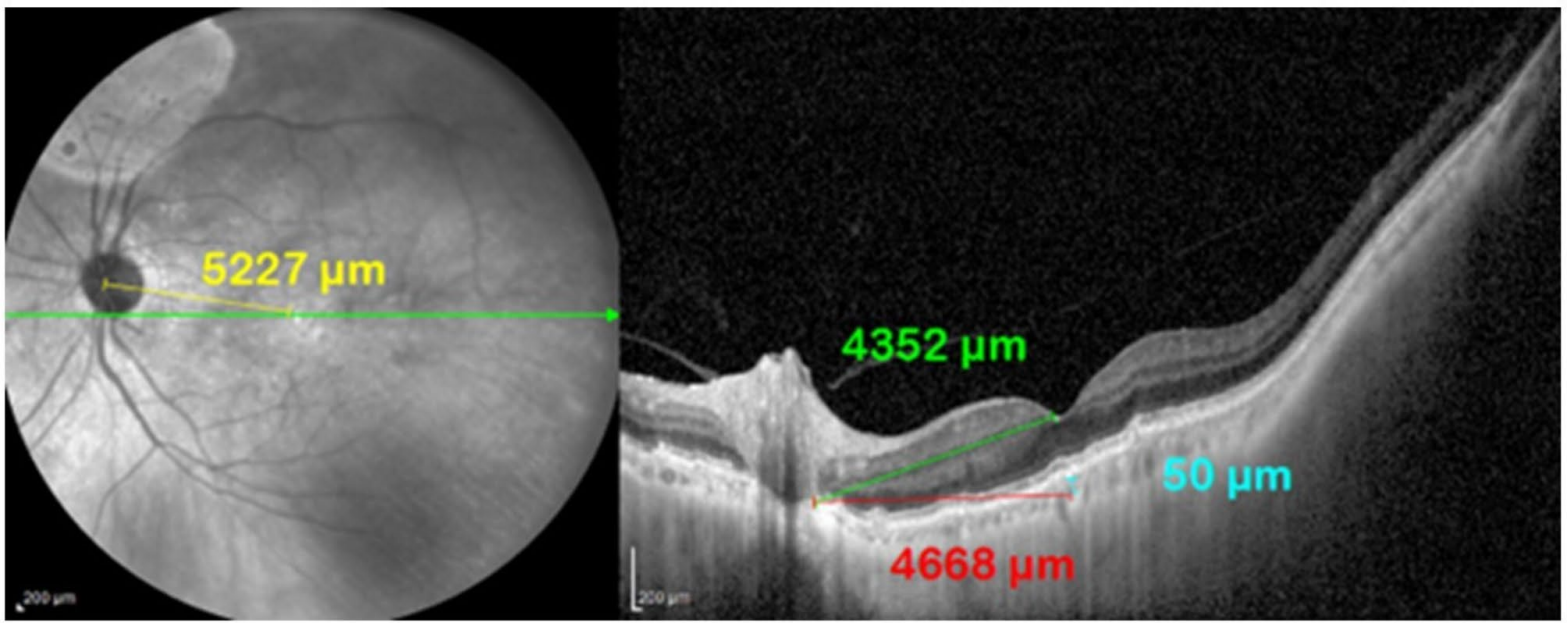
EDI-OCT image of the left posterior pole in a line scan mode and the corresponding topographic image using a wide-viewing system. The left photo demonstrates the topographic assessment of PFD 1 (yellow) using a line subtended from the bifurcation of the vessels of the optic disc to the fovea. The right photo demonstrates the tomographic assessment of PFD 2 (green) using a line subtended between the fovea and the end of the RPE layer at the optic disc. The right photo demonstrates the tomographic assessment of of PFD 3 (red) by subtending a line between a reference point (blue) in the choroid 50 μm from the posterior border of the sub-foveal RPE and the end of the RPE at the optic disc

PFD 1 is defined on a topographic fundus photo as the length of a line subtended from the bifurcation of the optic disc vessels to the fovea. The location of the fovea is determined on the topographic image using the foveal position on the corresponding tomographic scan as a reference. PFD 2 was defined on the tomographic image as the length of a line subtended from the fovea to the end of the RPE at the optic disc. PFD 3 was defined as the length of a line subtended from the choroidal reference point (a point in the choroid 50 μm from the posterior border of the sub-foveal RPE) to the end of the RPE at the optic disc. Figure 2.

### Statistical methodology

Data were analyzed using IBM SPSS version 27, with quantitative data presented as means, standard deviations, and ranges when parametric and median, and inter-quartile range (IQR) when non-parametric. Qualitative data were presented as numbers and percentages. We used the Chi-square test and/or Fisher exact test to compare groups regarding qualitative data. We used the independent t-test to compare two independent groups with quantitative data and parametric distribution and the Mann-Whitney test for non-parametric distribution. We used the One-Way ANOVA test to compare more than two groups regarding quantitative data and parametric distribution and the Kruskal-Wallis test for non-parametric distribution. The confidence interval was set at 95%. The p-value was considered significant when < 0.05.

**Fig. 3:**
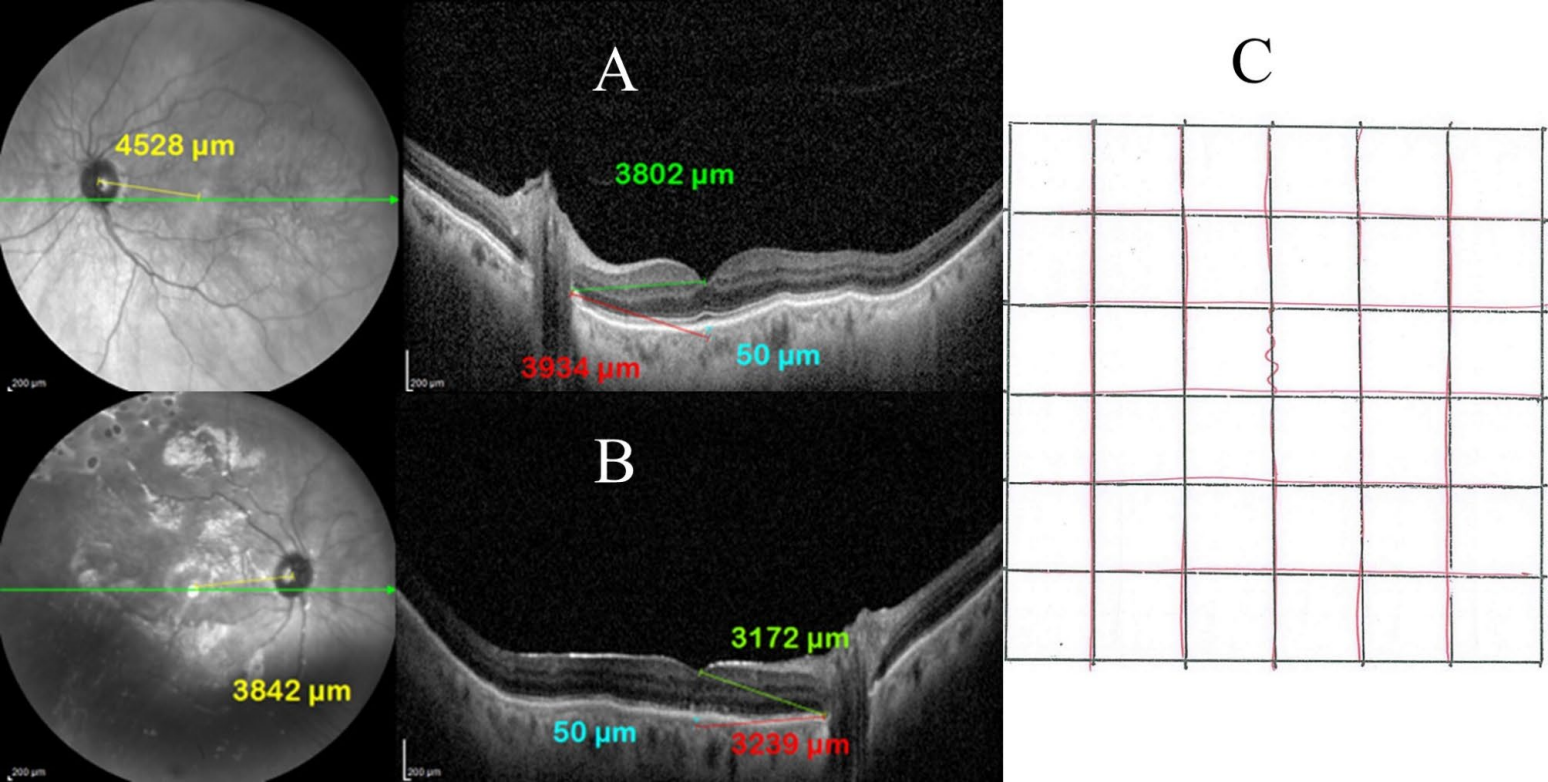
EDI-OCT image shows (**A**) the control eye and (**B**) the study eye of a 68-year-old male patient who had total RRD with PVR grade B in the right eye and a baseline visual acuity of hand movement (HM). He had successful retinal reattachment following PPV and silicone oil tamponade. In the control eye PFD 1 (yellow) measured 4528 μm, PFD 2 (green) measured 3802 μm, and PFD 3 (red) measured 3934 μm. In the study eye PFD 1 (yellow) measured 3842 μm, PFD 2 (green) measured 3172 μm, and PFD 3 (red) measured 3239 μm which indicated nasal displacement of the fovea. (**C**). A modified Amsler chart shows that the quantitative metamorphopsia score is 200. The patient had mild subjective metamorphopsia and a final BCVA of 0.3

**Fig. 4:**
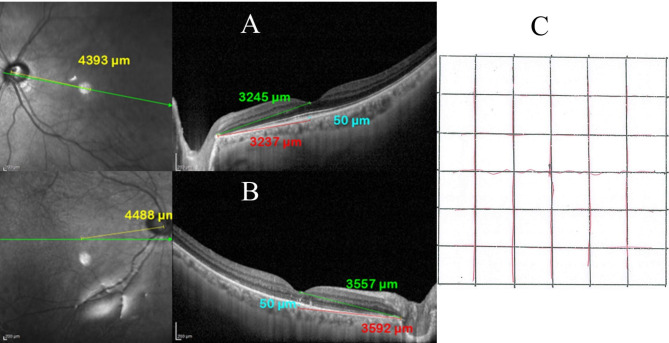
EDI-OCT image shows (**A**) the control eye and (**B**) the study eye of a 57-year-old male patient who had subtotal RRD with PVR grade A in the right eye and a baseline visual acuity of HM. He had successful retinal reattachment following PPV and silicone oil tamponade. In the control eye PFD 1 (yellow) measured 4393 μm, PFD 2 (green) measured 3245 μm, and PFD 3 (red) measured 3237 μm. In the study eye PFD 1 (yellow) measured 4488 μm, PFD 2 (green) measured 3557 μm, and PFD 3 (red) measured 3592 μm, which indicated temporal displacement of the fovea. (**C**). A modified Amsler chart shows that the quantitative metamorphopsia score is 1200. The patient had moderate subjective metamorphopsia and a final BCVA of 0.1

## Results

### Baseline characteristics of the study patients

The study included 100 eyes of 50 patients (50 study eyes and 50 control eyes). The male gender constituted 70% of patients (35 patients). The mean age was 53 years (range 12–82; SD 15). Twenty-two patients were phakic (44%), and 28 patients were pseudo-phakic (56%). The mean extension of the detachment was 3 quadrants (range 2–4; SD 0.73). Forty-one patients (82%) had a single break, and 9 patients (18%) had multiple breaks. Seventeen patients (34%) had PVR type A, 23 patients (46%) had PVR type B, and 10 patients (20%) had PVR type C. The mean baseline BCVA was 0.001 (decimal units). We used PFCL in all patients (100%), silicone oil (1000 cSt) in 46 patients (92%), and sulfur hexafluoride (SF6) gas in a non-expansile concentration (20%) in 4 patients (8%). Table 1.

**Table 1:** Baseline characteristics of the study patients

Baseline characteristic	Number (%)
Gender	
Male	35 (70)
Female	15 (30)
Age (years)	
<20	1 (2)
20–40	8 (16)
>40	41 (82)
Laterality	
OD	34 (68)
OS	16 (32)
Lens Status	
Phakic	22 (44)
Pseudophakic	28 (56)
Extension of the detachment (per quadrant involved; Q)	
2 Q	7 (14)
3 Q	17 (34)
4 Q	26 (52)
No. of breaks	
Single	41 (82)
Multiple	9 (18)
PVR	
Type A	17 (34)
Type B	23 (46)
Type C	10 (20)
Mean BCVA (decimal notation)	0.001

BCVA: best corrected visual acuity; No.: number; OD: oculus dexter; OS: oculus sinister; PVR: proliferative vitreoretinopathy; Q: quadrant

### Anatomical outcomes

#### Foveal shift

The incidence of foveal displacement was 70% (35 patients), of whom 24 patients (48%) had a nasal foveal shift, and 11 patients (22%) had a temporal foveal shift. The mean postoperative PFD 1 in the study eyes was 4450 μm (range 3223–5395 μm; SD 481). In the control eyes, the mean PFD 1 was 4638 μm (range 2929–5664 μm; SD 484). The mean post-operative foveal shift was 383 μm (range 4-1352; SD 299). The mean change in PFD 2 in the study eyes was 3572 μm (range 2516–4625 μm; SD 445). The mean PFD 2 of the control eyes was 3728 μm (range 2284–4862 μm; SD 453). The mean post-operative foveal shift measured 437 μm (range 37-1759; SD 335). The mean change in PFD 3 of the study eyes was 3575 μm (range 2464–4628 μm; SD 463). The mean PFD 3 in the control eyes was 3724 μm (range 2208–4852 μm; SD 479). The mean post-operative foveal shift measured 450 μm (range 8-1752; SD 351). (Figs 3, 4).

#### Foveal microstructural changes

The mean CMT was 250 μm; SD ± 36 (range 190–400 μm), VMI changes in the form of a globally-adherent ERM that did not the foveal contour developed in 23 cases (46%). DRIL occurred in 28 patients (56%). DROL occurred in 36 patients (72%), of whom 24 patients (48%) had partial DROL involving one or more outer retinal layers, and 12 patients (24%) had complete disruption of all outer retinal layers.

### Functional outcomes (BCVA and metamorphopsia)

The mean postoperative BCVA was 0.3 (SD 0.24; range 0.05–0.8). The assessment of metamorphopsia was possible only in 33 patients (66%) in whom BCVA was better than 0.1. The subjective metamorphopsia was mild in 13 patients (26%), moderate in 8 patients (16%), severe in 11 patients (22%), and very severe in 1 patient (2%). The mean total quantitative metamorphopsia was 587 mm (SD 406; range 100–1200). The mean vertical quantitative metamorphopsia was 349 mm (SD 236; range 50-1000), and the mean horizontal quantitative metamorphopsia was 341 mm (SD 217; range 40–800). Table 2.

**Table 2:** Anatomical and functional outcomes

Mean PFD shift (µ)	
- PFD 1	383
- PFD 2	437
- PFD 3	450
Foveal microstructural changes	
Mean CMT (µ)	250
ERM (no. of eyes; %)	23 (46)
DRIL (no. of eyes; %)	28 (56)
DROL	36 (72)
- Complete	12 (24)
- Partial	24 (48)
Mean post-operative BCVA (decimal notation)	0.3
Subjective metamorphopsia (33 patients); no. of eyes; %	
- Mild	13 (26)
- Moderate	8 (16)
- Severe	11 (22)
- Very severe	1 (2)
Mean quantitative metamorphopsia (33 patients) in mm	
- Total	587
- Vertical	349
- Horizontal	341

BCVA: best corrected visual acuity; CMT: central macular thickness; ERM; epiretinal membrane; DRIL: disorganized retinal inner layers; DROL: disorganized retinal outer layers; µ: micron; mm: millimeter; no.: number; OD: oculus dexter; OS: oculus sinister; PFD: papillo-foveal distance; PVR: proliferative vitreoretinopathy; Q: quadrant

### Statistical correlation of the studied parameters

#### Correlation between foveal shift and preoperative parameters

The statistical analysis revealed that PVR is a significant factor in foveal shift occurrence, (p 0.018). In contrast, the status of the crystalline lens, the number of retinal breaks, and the extension of retinal detachment are not significant contributing factors in the occurrence of foveal shift.

#### Correlation between foveal shift and postoperative outcome

The statistical analysis revealed that postoperative VMI and foveal microstructural changes (CMT, DRIL, DROL) are not significant factors in foveal shift occurrence. Table 3.

**Table 3:** Correlation between foveal shift, preoperative parameters, postoperative outcome

		Foveal shift	*p*-value
		Mean ± SD	
Lens status‡	Phakic	343 ± 291.5	0.333
	Pseudophakic	415 ± 306	
Number of breaks‡	Single	368 ± 286	0.544
	Multiple	452 ± 365	
Proliferative vitreoretinopathy‡‡	Type A	513 ± 296	0.018
	Type B	256 ± 209	
	Type C	456 ± 380	
VMI‡	Absent	371 ± 304	0.606
	Present	398 ± 299	
DRIL‡	Absent	394 ± 323	0.945
	Present	375 ± 285	
DROL‡‡	Absent	301 ± 214	0.327
	Partial	431 ± 350	
	Complete	384 ± 274	
Extension of retinal detachment	-		0.212
CMT	-		0.814

CMT, central macular thickness; DRIL, disorganized retinal inner layers; DROL, disorganized retinal outer layers; SD, standard deviation; VMI, vitreomacular interface

p-value < 0.05; ‡, Mann Whitney test; ‡‡, Kruskal Wallis test

#### Correlation between metamorphopsia and preoperative parameters

The statistical analysis revealed that the grade of PVR, the number of breaks, the extent of retinal detachment, and the status of the crystalline lens are not significant contributing factors in the occurrence of metamorphopsia. Nevertheless, we detected a trend toward an association between less metamorphopsia and less severe PVR, in the sense that mild metamorphopsia occurred more frequently in association with grade A PVR and severe metamorphopsia occurred more commonly with grade C PVR.

#### Correlation between metamorphopsia and postoperative outcome

The statistical analysis revealed that DROL was a significant factor in determining subjective metamorphopsia, (p 0.012). Complete and partial DROL were more associated with severe metamorphopsia and absent DROL with mild metamorphopsia. There was a statistically significant difference between subjective metamorphopsia and quantitative metamorphopsia (p 0.012). In contrast, the statistical analysis revealed that foveal shift, CMT, DRIL, and VMI changes did not correlate significantly with metamorphopsia. Tables 4 and 5.

**Table 4:** Correlation between metamorphopsia, preoperative parameters, postoperative outcome

		Metamorphopsia			*p*-value
		Mild	Moderate	Severe	
		No. = 13	No. = 8	No. = 12	
Lens status*	Phakic	6 (46%)	3 (37.5%)	4 (33%)	0.8
	Pseudophakic	7 (54%)	5 (62.5%)	8 (67%)	
Number of breaks*	Single	13 (100%)	6 (75%)	10 (83%)	0.2
	Multiple	0 (0%)	2 (25%)	2 (17%)	
Extension of the detachment ⁑	Mean ± SD	3.31 ± 0.85	3.38 ± 0.74	3.25 ± 0.87	0.9
	Range	2–4	2–4	2–4	
PVR*	Type A	7 (54%)	2 (25%)	4 (33%)	0.65
	Type B	4 (31%)	5 (62.5%)	6 (50%)	
	Type C	2 (15%)	1 (12.5%)	2 (17%)	
CMT⁑	Mean ± SD	264 ± 52	246 ± 30	239 ± 23	0.3
VMI*	Absent	7 (54%)	5 (62.5%)	9 (75%)	0.5
	Present	6 (46%)	3 (37.5%)	3 (25%)	
DRIL*	Absent	9 (69%)	3 (37.5%)	6 (50%)	0.3
	Present	4 (31%)	5 (62.5%)	6 (50%)	
DROL⁑	Absent	8 (61.5%)	4 (50%)	0 (0%)	0.012
	Partial	4 (31%)	3 (37.5%)	6 (50%)	
	Complete	1 (8%)	1 (12.5%)	6 (50%)	

CMT, central macular thickness; DRIL, disorganized retinal inner layers; DROL, disorganized retinal outer layers; PVR, proliferative vitreoretinopathy; SD, standard deviation; VMI, vitreomacular interface

p-value < 0.05; * Chi-square test; ⁑ One Way ANOVA test

**Table 5:** Correlation between subjective and quantitative metamorphopsia

		Subjective Metamorphopsia			
		Mild	Moderate	Severe	*p* value
		No. = 13	No. = 8	No. = 12	
Quantitative Metamorphopsia⁑	Mean ± SD	347 ± 315.5	831 ± 345	683 ± 417	0.012
	Range	100–1200	300–1200	250–1200	

*p* < 0.05; ⁑One Way ANOVA test

#### Correlation between postoperative BCVA versus foveal shift, foveal microstructural changes, and metamorphopsia

The statistical analysis revealed no significant correlation between final BCVA, foveal displacement, microstructural changes, or metamorphopsia.

## Discussion

Metamorphopsia following retinal detachment surgery affects many patients despite successful retinal reattachment and improvement of BCVA. The proposed mechanisms for metamorphopsia are foveal displacement and the development of foveal microstructural changes. In the present study, foveal displacement occurred in 70% of cases. Our results are comparable to Shiragami et al. [12], Codenotti et al. [13], Lee et al. [14], Cobos et al. [15], who reported retinal displacement in 57–70% of cases. Conversely, Mahmoudzadeh et al. [16], dell’Omo et al. [17], Brosh et al. [18], Chelazzi et al. [19], and Kumar et al. [20] documented lower rates, between 6.4% and 35.2%. These discrepancies may be attributed to variations in surgical techniques, grading methodologies, or imaging modalities. Shiragami et al. [12], Codenotti et al. [13], and Cobos et al. [15] used fundus autofluorescence (FAF) for documenting retinal vessels position and displacement. In comparison, in the present study, we used the method described by Datlinger et al. [21], and Nair et al. [22], to detect postoperative foveal displacement. It consisted of estimating the change in the position of the fovea in the horizontal meridian in relation to the optic disc. To accurately measure the PFD, we used the bifurcation of the large veins at the ONH as the starting point of the line scan rather than the edge of the disc to avoid fallacies in measurements due to peripapillary atrophy and enlarged beta zone. In a study by Ishida et al. [23], the authors mentioned that the displacement of the fovea might not be the same as the displacement of the retinal vessels or the superficial retina. Accordingly, in the present study, we included measurement of the PFD on the tomographic image in relation to a reference point in the subfoveal choroid (PFD3). In the present study, foveal displacement did not correlate significantly with metamorphopsia. Similarly, Kumar et al. [20], found no relationship between foveal displacement and subjective metamorphopsia in a study that included 39 patients with successful RRD repair. Guber et al. [24] found that metamorphopsia persisted despite decreased displacement during the follow-up period in a study of nine patients with retinal shift after RRD repair by PPV. In contrast, Chelazzi et al. [19] and Lee et al. [14] reported that retinal displacement was a significant factor in determining metamorphopsia following fovea-involving RRD repaired by PPV. Conversely, Brosh et al. [18] mentioned that the retinal displacement was significantly related to metamorphopsia in a study of 238 patients of RRD treated with pneumatic retinopexy or PPV. DROL following RRD surgery is related to retinal stretch produced by the intraocular tamponade and complete drainage of the subretinal fluid. Retinal stretch produces disintegration of the photoreceptors and their interdigitations. Furthermore, the localized displacement of a group of photoreceptors relative to the remainder of the retina leads to a shift in the visual field projection of the affected photoreceptors. In the presence of normal binocular vision before the onset of the disease, this unilateral displacement of photoreceptors will lead to loss of visual field correspondence with the cortically paired photoreceptor element in the fellow eye. The resultant discrepancy between the real and perceived visual field projection can induce metamorphopsia [25, 26]. In our study, partial or failed restoration of outer retinal layers (DROL) was observed in 72% of cases, with DROL significantly associated with metamorphopsia. The grade of metamorphopsia increased with the degree of DROL, in the sense that complete and partial DROL correlated with severe metamorphopsia, while absent DROL correlated with mild metamorphopsia. In contrast, other authors reported lower values of DROL following successful RRD repair, ranging from 14.8 − 33% of cases [18, 27–29]. Brosh et al. [18] and Muni et al. [29] stated that DROL was significantly associated with retinal displacement after successful RRD repair. PVR poses a significant challenge in RRD surgery outcomes. There is a scarcity of research on the relationship between PVR and retinal displacement. Many studies excluded cases with PVR, particularly advanced PVR [12, 18, 19, 30–32] Our findings indicate that varying grades of PVR correlated significantly with foveal displacement. Conversely, Filippelli et al. [33], investigated post-PPV retinal displacement in PVR cases and concluded that the severity of PVR did not affect the likelihood of retinal displacement. There are limited reports in the literature on the relationship between subjective or quantitative metamorphopsia and different grades of PVR. In our study, PVR did not correlate significantly with metamorphopsia. Similarly, Saleh et al. [34] concluded that metamorphopsia frequently occurs after RRD surgery independent of the PVR stage. In the present study, we found that preoperative parameters such as the number of retinal breaks, the status of the crystalline lens, and the extent of retinal detachment are not significant factors in the development of postoperative foveal displacement or metamorphopsia. Similarly, dell’Omo et al. [32] and Filippelli et al. [33] found no significant effect of retinal breaks on retinal displacement. Yamada et al. [35] concluded that the extent of RRD was not significantly related to the degree of metamorphopsia in a study of 77 patients with successful retinal re-attachment after PPV. Cobos et al. [15], Schawkat et al. [31], dell’Omo et al. [32], and Casswell et al. [36] found no significant impact of detachment extent on foveal displacement. Conversely, Kumar et al. [20] found that subtotal detachment significantly influenced postoperative metamorphopsia. Shiragami et al. [12] and Codenotti et al. [13] reported significant associations between RRD extent and postoperative retinal displacement. In the present study, our results showed significant improvement in BCVA, p 0.01. The mean postoperative BCVA was 0.3 compared with the baseline mean BCVA of 0.001. We found that postoperative foveal displacement, metamorphopsia, and foveal microstructural changes did not correlate significantly with final BCVA. These findings are consistent with Cobos et al. [15], Chelazzi et al. [19], and dell’Omo et al. [32], who reported no significant impact of retinal displacement on final BCVA. In contrast, Mahmoudzadeh et al. [16] and Casswell et al. [37] found worse BCVA outcomes associated with retinal displacement. Wakabayashi et al. [38], Kobayashi et al. [39], and Chatziralli et al. [40] found a significant correlation between postoperative BCVA and foveal microstructural restoration in macula-off RRD patients. Tanikawa et al. [41] found no significant relation between postoperative BCVA and metamorphopsia in patients who had PPV for idiopathic ERM removal. Conversely, Fukuyama et al. [42] found a significant relationship between quantitative metamorphopsia and final BCVA in ERM, MH, and RRD cases. The different factors influencing both parameters could explain the disparity between visual acuity and metamorphopsia. Visual acuity could decrease due to distortion of the photoreceptors, mechanical strain, intraretinal edema, and obstruction of the axoplasmic flow [43]. In contrast, metamorphopsia could develop due to interference with 1-to-1 correspondence between the retinal image and the nervous system, insufficient synaptic junctions, or low photoreceptor sensitivity [44]. Our results showed an overt disparity between quantitative and subjective methods for the assessment of metamorphopsia, (p 0.012), in the sense that a given patient could have a good score in subjective testing and a poor one on objective assessment. This dictates adherence to a single method for the initial assessment of metamorphopsia and during follow-up visits. We suggest using the subjective method since it is more related to the patient’s perception and is more practical during examination in the clinic. In the present study, our results showed that there are certain factors associated with RRD that represent a high-risk profile for developing post-operative foveal displacement and metamorphopsia after successful reattachment. The presence of DROL, and moderate or severe PVR are associated with a higher risk of foveal displacement and metamorphopsia. The present study has several limitations. Firstly, we used SO in the majority of cases, and PFCL in all cases. Hence, there is no concurrent comparison between the effect of silicone oil and other tamponade agents and the use of PFCL on foveal displacement. Secondly, in the present study, we used a single technique, which is PPV to treat all cases of RRD. Consequently, there is no concurrent comparison with the outcome of other surgical techniques for RRD. Thirdly, the sample size recruited for the study was relatively small and included aspects of inhomogeneity at baseline including the causality of cases with different grades of PVR and the presence of single or multiple retinal breaks; which could be a source of bias during statistical analysis and could explain the statistically insignificant outcomes in some of the parameters tested. Fourthly, the topographic and tomographic measurements of foveal displacement are performed manually with possible intra- and inter-operator variations in measurements. Finally, although we used check points, namely, PFD1, PFD 2, and PFD 3 to ensure accurate assessment of foveal displacement, our method lacked comparison with other modalities that could detect foveal displacement such as FAF. We recommend performing future studies that include a larger number of patients, and concurrent comparison groups comparing different tamponade agents, different surgical techniques, and different techniques for assessment of foveal displacement based on randomization.

## Conclusion

Foveal displacement and metamorphopsia after successful retinal reattachment pose significant morbidity. Identification of high-risk patients who are prone to develop post-operative foveal displacement and metamorphopsia is important for preoperative patient counseling and patient compliance with visual rehabilitation after surgery. UHR-OCT is a fundamental tool in the postoperative evaluation of the anatomical outcome after successful retinal re-attachment surgery and its relation to visual function.

## Data Availability

The data that support the findings of this study are available on request from the corresponding author (ML). Access to this data will be granted exclusively to researchers of entities who meet the criteria for access to confidential data.
